# Stem Cells, Hematopoiesis and Lineage Tracing: Transplantation-Centric Views and Beyond

**DOI:** 10.3389/fcell.2022.903528

**Published:** 2022-04-27

**Authors:** Anna Konturek-Ciesla, David Bryder

**Affiliations:** Division of Molecular Hematology, Lund University, Lund, Sweden

**Keywords:** lineage tracing, adult hematopoiesis, hematopoietic stem cells, bone marrow transplantation, genetic barcoding

## Abstract

An appropriate production of mature blood cells, or hematopoiesis, is essential for organismal health and homeostasis. In this developmental cascade, hematopoietic stem cells (HSCs) differentiate into intermediate progenitor types, that subsequently give rise to the many distinct blood cell lineages. Here, we describe tools and methods that permit for temporal and native clonal-level HSC lineage tracing in the mouse, and that can now be combined with emerging single-cell molecular analyses. We integrate new insights derived from such experimental paradigms with past knowledge, which has predominantly been derived from transplantation-based approaches. Finally, we outline current knowledge and novel strategies derived from studies aimed to trace human HSC-derived hematopoiesis.

## Introduction

Hematopoiesis refers to the highly regulated processes in which individual blood cells are formed. In steady state, the different blood cell lineages vary considerably in terms of both lifespans and numbers. For instance, in murine peripheral blood (PB), erythrocytes have a lifespan of approximately 40 days (Van Putten and Croon, 1958) and a frequency of approximately 8 × 10^9^ cells/ml, while most circulating mature neutrophils have an estimated lifespan of less than a day (Pillay et al., 2010) but are >5,000-fold less abundant than erythrocytes. B cells, by contrast, are slightly more numerous than neutrophils but with considerably longer average lifespans; naive B cells have an estimated half-life of 13–22 weeks, while memory B cells can persist for >2 years (Jones et al., 2015).

Despite the fundamental differences in generation rates of different blood cell lineages, most hematopoiesis originates from rare bone marrow (BM) hematopoietic stem cells (HSCs). Disruption of hematopoiesis in response to molecular insults or due to environmental stressors can result in benign or malignant hematologic disorders. Therefore, considerable efforts have been devoted to reveal the cellular differentiation pathways of HSCs, which along with self-renewal represents the primary functional traits of HSCs. While HSC biology today represents a well-established research field, recent technological advances have begun to challenge concepts traditionally linked to HSC function. Here, we have tried to describe the key features of such emerging data, and to place it in the context of prior paradigms.

## Assaying Hematopoietic Stem Cell Function: Transplantation

Transplantation allows for tracking multilineage cell differentiation *in vivo* and in some embodiments also to assess HSC self-renewal and has for long been the prevailing method to assess HSC function. This typically involves injection of candidate HSCs into conditioned recipients, followed by monitoring the fate of the transplanted cells. Because a continuous long-term production of mature blood cells is a key property of HSCs, transplantation in its simplest form provides qualitative information on HSC function over time. Furthermore, self-renewal and/or exhaustion can be assessed by serial transplantation. When performed in a competitive setting, transplantation allows quantification of the repopulating activity of the cells evaluated (Busch and Rodewald, 2016).

**FIGURE 1:**
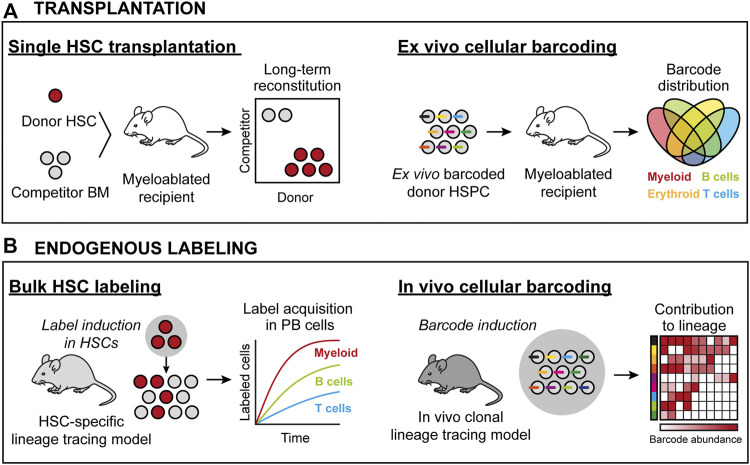
HSC lineage tracing techniques in the mouse. **(A)** Transplantation-based approaches. *Left:* Transplantation of prospectively isolated candidate HSCs permits for assaying HSC activity at the single cell level. Here, all donor progeny in the mouse is derived from one cell. *Right:* Transplantation of *ex vivo* barcoded HSCs allows for monitoring the frequency of individual barcodes/clones from a population of HSCs. Here, donor progeny is derived from multiple HSCs, with the clonality assigned retrospectively by the identity and frequency of individual barcodes in defined cell subsets. **(B)** Approaches to assess native HSC activity. *Left:* Using genetic drivers, irreversible recombination events are induced that permit the tracing of a heritable donor marker. Here, the contribution from a pool of HSCs is measured in defined progenitors based on the induced donor marker. *Right:* Endogenous clonal HSC lineage tracing. Here, the ability to induce unique donor markers in each cell permits for the clonal assessments similar to as in **(A)**.

The differentiation potential and frequency of candidate HSCs can be measured using “bulk” transplantations (e.g., the transplantation of several cells) (Spangrude et al., 1988; Smith et al., 1991; Hu and Smyth, 2009). Bulk approaches typically do not require advanced cell purification strategies and can be performed using relatively few animals. However, these procedures only provide information on the average function and behavior of the population of transplanted HSCs. For the purpose of assessing clonal HSC behavior, the single-cell transplantation assay was developed (Smith et al., 1991; Osawa et al., 1996) (Figure 1A). This was made possible *via* advances in HSC isolation strategies, a necessity due to the rarity of HSCs (estimated to only 0.005% of all BM cells). Based on differential expression of surface markers, long-term (LT) HSCs were identified as a minor fraction of Lineage- Sca1+ cKit+ (LSK) BM cells, that can be further enriched using additional markers such as CD48 (Kiel et al., 2005), CD150 (Slamf1) (Kiel et al., 2005), CD34 (Osawa et al., 1996), CD135 (Flt3) (Adolfsson et al., 2001; Christensen and Weissman, 2001), CD105 (Endoglin) (Chen et al., 2002) and CD201 (Epcr) (Balazs et al., 2006). While transplantable HSC activity can be further enriched using additional markers, a current consensus is that HSC are phenotypically defined as LSK CD135- CD48- CD150+ (Challen et al., 2021), with hematopoietic stem and progenitor cells (HSPCs) downstream of HSCs presenting with alternative marker combinations.

A prevailing method to track HSC-derived hematopoiesis in the transplantation setting employs congenic C57BL/6 mouse strains with different *Ptprc* alleles (commonly known as CD45.1 or CD45.2), where one strain is used as the host (recipient) and the other for the cells to be functionally assessed. This allows evaluation of donor reconstitution in PB leukocyte populations, but precludes determination of chimerism in mature platelets and erythrocytes, which lack expression of CD45. Tracking HSC contribution to these latter lineages was later made possible by the development and implementation of transgenic mouse strains in which HSCs and their progeny ubiquitously express a fluorescent protein (Sanjuan-Pla et al., 2013; Yamamoto et al., 2013). A fundamental insight from large-scale single HSC transplantation studies is that single HSC can regenerate the full spectra of PB cells as well as new HSCs (Osawa et al., 1996; Dykstra et al., 2007). Single-cell transplantation data have also revealed that individual HSC clones can contribute to hematopoiesis for different time periods (Osawa et al., 1996; Morrison et al., 1997; Yang et al., 2005), suggesting variable self-renewal potential (Ema et al., 2005). Importantly, even within the phenotypically most primitive HSC pool, individual HSCs display vastly heterogenous kinetics and magnitudes of reconstitution that have been reported to range from 0.1% from some candidate HSCs up to 80–90% chimerism from others (Müller-Sieburg et al., 2002; Muller-Sieburg et al., 2004; Dykstra et al., 2007; Morita et al., 2010; Yamamoto et al., 2013). This knowledge has strong implications also for the design and interpretation of bulk transplantation experiments.

Another key observation from transplantation studies has been that not all phenotypic HSCs contribute equally to the different blood cell lineages. In fact, only a small fraction of candidate HSCs is capable of robust multilineage hematopoiesis. Instead, the majority of HSCs display preferential (biased) differentiation towards certain lineages. This was first demonstrated for the myeloid and lymphoid lineages (Müller-Sieburg et al., 2002; Muller-Sieburg et al., 2004; Dykstra et al., 2007), and later for the platelet lineage (Sanjuan-Pla et al., 2013; Yamamoto et al., 2013; Carrelha et al., 2018). Prospective enrichment of different lineage-biased HSCs were reported based on the surface expression of CD150 (Beerman et al., 2010), CD41 (Gekas and Graf, 2013), CD61 (Mann et al., 2018) or Neogenin-1 (Gulati et al., 2019), using genetic reporter models (Sanjuan-Pla et al., 2013), or by differential dye efflux activities (Challen et al., 2010). In addition, lineage-biased HSCs were suggested to possess distinct regulatory programs, as evidenced by their differential responses to cytokines [such as interleukin-7 (Muller-Sieburg et al., 2004) or transforming growth factor b (Challen et al., 2010)] and inflammatory signals (Matatall et al., 2014; Chavez et al., 2022), distinct transcriptomic signatures (Mann et al., 2018; Gulati et al., 2019) and/or preferential occupancy of specific BM niches (Pinho et al., 2018). HSC heterogeneity should have direct implications for blood cell production during homeostasis or in response to stress, and has also been reported to be altered during ontogeny. Accordingly, while multipotent and lineage-biased HSCs are present throughout fetal and adult life, their relative abundance shifts during development, with a steadily declining representation of HSC clones with robust lymphoid differentiation capacity (Benz et al., 2012). This redistribution of compartments further exacerbates during physiological aging and results in relative dominance of myeloid-biased clones (Muller-Sieburg et al., 2004; Cho et al., 2008; Beerman et al., 2010; Dykstra et al., 2011; Benz et al., 2012). Additional studies have emphasized an age-associated increase in platelet-biased HSCs (Grover et al., 2016) and myeloid-restricted progenitors within the phenotypic HSC compartment (Yamamoto et al., 2018).

Altogether, (single cell) transplantation studies have suggested that individual HSCs are heterogenous as for their lineage output, durability of engraftment, self-renewal potential and responses to external stimuli. Clonal HSC diversity might represent a mechanism that ensures appropriate responses during emergency hematopoiesis. At the same time, heterogeneity of stemness phenotypes is likely relevant also to malignant transformation, in which selective expansion of clones with the most competitive attributes is observed (Jaiswal and Ebert, 2019).

## Assaying Hematopoietic Stem Cell Function: Cellular Barcoding and Gene Expression Profiling

Although single cell transplantations assay the function of individual HSCs, the methodology associates with several caveats, including laborious and time-consuming analyses and limited data output (e.g., information from few cells). This associates with high costs and ethical considerations. Moreover, because of detection limits, somewhat arbitrary reconstitution cutoff values are often applied to define the reconstituted animals. Therefore, alternative lineage tracing techniques based on genetic barcoding have emerged and undergo continuous refinement.

In its simplest form, genetic barcoding involves the labeling of individual HSPCs with unique and heritable DNA tags (barcodes), typically delivered by stable-integrating recombinant viral approaches. Next, the barcoded cells are transplanted, which enable the analysis of overlap and the unique distribution of barcodes across different hematopoietic lineages and over time. Given the complexity of these barcoding libraries, they allow for simultaneous analysis of multiple individual clones in a single recipient (Figure 1A).

Pioneering attempts to genetically barcode HSCs involved transplantation of retroviral-marked candidate HSCs, followed by Southern blot analyses (Lemischka et al., 1986; Jordan and Lemischka, 1990). These studies revealed hematopoietic progenitor classes with biased differentiation output (Lemischka et al., 1986) and suggested that long-term reconstitution associates with relatively few HSC clones (Jordan and Lemischka, 1990). Subsequent technological advances (e.g., readout *via* high-throughput sequencing) have allowed for more sensitive detection. While initially used to study T cell biology (Schepers et al., 2008; van Heijst et al., 2009), sequencing-based barcoding methods were rapidly adapted to HSC biology (Gerrits et al., 2010; Lu et al., 2011) to precisely quantify HSC contribution to post-transplantation hematopoiesis, to assess functional HSC heterogeneity and to establish clonal relationships between HSPCs and their mature progeny (Lu et al., 2011). Later studies have demonstrated that the behavior of single HSCs can be influenced by the number of injected cells (Brewer et al., 2016), the presence of HSPCs with defective differentiation (Nguyen et al., 2018) and also by the conditioning regimen (Lu et al., 2019).

Cellular barcoding has also been applied to reveal functional changes of HSCs during aging (Verovskaya et al., 2013; Wahlestedt et al., 2017). It has been shown that aging shifts the clonal composition of the HSC pool, wherein myeloid-restricted clones appear more abundant at the expense of clones contributing to T-cell production. At the same time, the magnitude of differentiation of individual aged HSCs was lower than their younger counterparts (Wahlestedt et al., 2017). This suggests that aging not only drives changes in clonal diversity of the HSC compartment, but also differentially affects the function of individual HSCs. Similar observations were reported in rhesus macaques after autologous transplantation of *ex vivo* barcoded HSPCs; lineage restricted HSPCs increased, but the total number of actively contributing HSC clones progressively declined with age (Yu et al., 2018). Other lineage tracing studies using primate transplantation models have supported that HSPC differentiation is heterogenous at the single cell level (Kim et al., 2014; Wu et al., 2014; Koelle et al., 2017). Importantly, these studies have also described a common pattern of post-transplantation hematopoiesis, in which an early reconstitution phase associates with a robust contribution from short-term progenitors. This is followed by a gradual switch towards more stable hematopoiesis from multipotent HSCs in the long-term (Kim et al., 2014; Wu et al., 2014; Koelle et al., 2017).

Most recently, cellular barcoding and high-throughput mRNA profiling have been combined, with the objective to simultaneously try to relate HSC differentiation histories (fates) to transcriptional signatures (states) (Rodriguez-Fraticelli et al., 2020; Weinreb et al., 2020). The principal assumption here is that daughter cells are alike and thus, that the transcriptomic information acquired from one cell can serve as a proxy for its’ sister, for which the fate is independently determined. In its’ initial implementation, it was observed that transcriptomic information alone was insufficient to predict cell potential and suggested that additional regulatory circuits (i.e., epigenetic states) likely play a contributiong role in fate determination of HSCs (Weinreb et al., 2020). In other work, two fractions of “low-” and “high-output” HSCs were identified that presented with differences in their differentiation activity, long-term repopulation, and transcriptomic signatures. Interestingly, the most potent HSC clones were found to generate low progeny output and their molecular signature highly overlapped with megakaryocyte-lineage priming (Rodriguez-Fraticelli et al., 2020).

## Assaying Hematopoietic Stem Cell Function: *In Vivo* Lineage Tracing

While HSCs can regenerate an entire hematopoietic system following transplantation, it is now well recognized that this might reflect poorly of the dynamics and physiology of native hematopoiesis. For instance, the requirement for harsh cytotoxic conditioning (total body irradiation)—needed to promote HSC engraftment—has a range of systemic effects that are likely to influence the fate of transplanted cells. Specifically, damage to the BM microenvironment/niches may have a non-physiologic impact on the behavior of transplanted HSCs that occupy such a niche. For this reason, alternative conditioning protocols that exploit monoclonal antibodies to specifically deplete cell populations of interest have emerged, and which as such may present a more physiologic setting with less undesired side effects. Using purified antibodies or antibody-drug conjugates, Czechowicz and others demonstrated that antibody-mediated conditioning efficiently depletes endogenous HSPCs and allows for robust and long-term engraftment of transplanted HSCs (Czechowicz et al., 2007; Palchaudhuri et al., 2016; Czechowicz et al., 2019). Some evidence indicates differential clonal dynamics and donor HSPC reconstitution patterns in mice subjected to irradiation and less invasive antibody-based conditioning (Säwen et al., 2018; Lu et al., 2019). Apart from the conditioning regimen, the dose of transplanted cells may also impact on the clonal dynamics of hematopoietic regeneration (Brewer et al., 2016). Finally, *in vitro* manipulations prior to transplantation might select for specific HSC clones or introduce bias in their lineage output. Therefore, methods to label HSCs *in situ* and to track their progeny in more native contexts have been developed.

*In vivo* genetic lineage tracing allows for prospective tracking and quantification of HSC differentiation. They typically involve the use of transgenic or knock-in reporter mouse strains that exploit the activity of enzymes, such as Cre recombinase or Sleeping Beauty transposase, to induce irreversible HSC labeling with fluorescent proteins or DNA barcodes. As these labels are inherited by all progeny of HSCs, they allow for determination of the magnitude and kinetics of HSC differentiation and to infer cellular relationships. Moreover, if the systems are under the control of an inducible element, such as estrogen receptor ERT fused to Cre recombinase or the Tet-ON system, they enable the study of temporal events (Figure 1B).

Clonal analysis using an inducible transposon mobilization system suggested that HSCs contribute minimally to ongoing hematopoiesis, and rather that native hematopoiesis is supported by a highly polyclonal output from multipotent progenitors (MPPs) (Sun et al., 2014). This output was suggested to reflect the activity of distinct lineage restricted precursors that reside within the MPP compartment (Rodriguez-Fraticelli et al., 2018)—an interpretation in line with previous transplantation and gene expression studies (Paul et al., 2015; Pietras et al., 2015; Velten et al., 2017). Using an alternative lineage tracing model, Busch and others similarly observed a low contribution of LT-HSC to native hematopoiesis (Busch et al., 2015). In this latter study, HSC specific labelling was achieved *via* an inducible Cre-mediated recombination in Tie2-expressing cells that drives the expression of a fluorescent reporter in only a small fraction (<1%) of phenotypic HSCs, but that leads to all hematopoietic lineages (but not all hematopoietic cells) acquiring the fluorescent label in the long-term. At the same time, the HSC flux to the immediate short-term HSCs (ST-HSCs, in other studies referred to as MPPs) was extremely rare, suggesting that also ST-HSCs need to self-renew to maintain their numbers and differentiation potential (Busch et al., 2015). Because enhanced proliferation and perhaps less cytoprotection of HSPCs downstream of HSCs might associate with a higher rate of accumulating DNA mutations, an intriguing hypothesis was laid forward in which a slow but continuous input from the most primitive HSCs might act as a rare source of new clones that serve to “rejuvenate” progenitor pools and thereby prevent malignant transformation (Dorshkind et al., 2020).

Additional genetic lineage tracing studies further supported an important role for HSCs in native hematopoiesis. Using an Fgd5-based system, we showed that HSCs continuously supply all major hematopoietic lineages throughout life (Säwen et al., 2018). In line with other reports using Pdzk1ip1- (Sawai et al., 2016; Upadhaya et al., 2018) or Krt18- (Chapple et al., 2018) based labeling systems, we observed that platelet generation occurred with the fastest kinetics and was followed by the emergence of myeloid and erythroid lineages. In contrast, the corresponding fractions of labeled lymphocytes were produced with substantially slower kinetics, with a decline in HSC differentiation potential during aging (Säwen et al., 2018). While the definitive mechanisms for age-associated HSC deterioration remains to be determined, the loss of HSC clones competent at producing multilineage output has been proposed not only using cellular barcoding (Verovskaya et al., 2013; Wahlestedt et al., 2017), but also more recently by HSC lineage tracing using a Confetti reporter system (Ganuza et al., 2019).

An exciting new development combines genetic lineage tracing with single cell transcriptome profiling, which allows for simultaneous interrogation of cell fates and molecular attributes (Bowling et al., 2020; Pei et al., 2020). The *PolyloxExpress* model exploits Cre-mediated recombination to create a unique set of DNA barcodes, subsequently transcribed as mRNA (Pei et al., 2020). By contrast, the CARLIN (CRISPR array repair lineage tracing) system generates expressible barcodes by CRISPR/Cas9-mediated mutagenesis with an array of gRNA target sites (Bowling et al., 2020). Using these approaches, multipotent and differentiation-inactive (or “childless”) HSCs were identified, whose fates were reported to correlate highly with a distinct transcriptional signature. Furthermore, using the CARLIN model, it was proposed that myeloablative stress induces restricted responses from only a few HSC clones, which appears to be in line with previous reports on oligoclonal hematopoiesis in irradiated and antibody-conditioned recipients post transplantation (Czechowicz et al., 2019). These intriguing observations suggest that HSC lineage specification, differentiation bias or specific clonal responses to hematopoietic challenges are (epi)genetically inscribed, and can be predicted at least to some extent by molecular profiling (Yu et al., 2017).

## Assaying Hematopoietic Stem Cell Function: The Human System

For obvious reasons, studies of native human hematopoiesis represent a particular challenge. Therefore, much effort has focused on development of (murine) xenograft systems permissive for human HSPCs engraftment (Doulatov et al., 2012). Pioneering work exploited mice with a spontaneous mutation (*Prkdc* ^scid^) that leads to an absence of functional murine B and T cells. The SCID model was successfully used to engraft human PB leukocytes (Mosier et al., 1988), fetal tissues (McCune et al., 1988), and BM cells (Kamel-Reid and Dick, 1988; Lapidot et al., 1992). Further refinement of recipient strains involved backcrossing of the *Prkdc* ^scid^ mutation onto the nonobese diabetic (NOD) strain (Shultz et al., 1995). NOD mice harbor a genetic variant of the *Sirpa* gene that cross-reacts with human CD47, leading to inhibition of macrophage-mediated phagocytosis (Takenaka et al., 2007). Finally, to prevent NK cell-mediated immunity, NOD-SCID mice with a deleted IL-2 receptor common gamma chain (*Il2rg−/−*) were generated (Shultz et al., 2005). Consequently, the resulting NOD-SCID *Il2rg−/−* (NSG) recipients show combined B, T, and NK cell deficiency, with host macrophage tolerance. Importantly, the NSG strain is more permissive for human HSPC engraftment and is resistant to development of thymic lymphomas often emerging in NOD-SCID mice (Doulatov et al., 2012). Humanized mouse systems have been continuously improved to allow for more efficient engraftment, tolerance, and differentiation of human HSPCs, and has been extensively reviewed in (Martinov et al., 2021).

**FIGURE 2:**
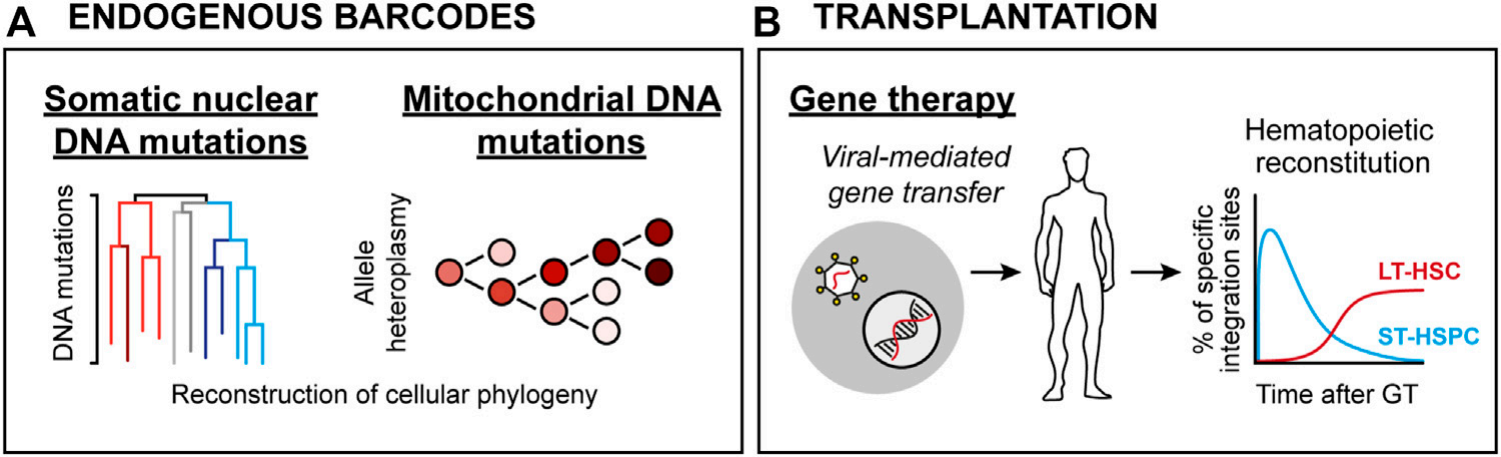
HSC lineage tracing techniques in humans. **(A)** Endogenous clonal HSC lineage tracing. Here, defined blood cell types are interrogated for either spontaneous nuclear mutation patterns (*Left*) or mitochondrial DNA heteroplasmy (*Right*), allowing for retrospective phylogenic reconstruction and quantification of clonal contributions. **(B)** Clonal assessments based on viral integration sites in patients subjected to viral-mediated gene therapy. Here, the integration sites of therapeutic vectors (which integrate randomly throughout the genome) are determined and used as barcodes.

Despite significant advancements, the murine xenotransplantation systems associate with several limitations, including overall low-level HSPC engraftment, ineffective production of human erythrocytes, platelets and peripheral neutrophils, xenogeneic graft-to-host responses and relatively short lifespans (Martinov et al., 2021). Therefore, human HSC function has been approached by analysis of naturally occurring genetic alterations, including shifts in X chromosome inactivation pattern (Catlin et al., 2011), average telomere length distribution (Werner et al., 2015) or more recently, the tracing of somatic DNA mutations (Lee-Six et al., 2018; Osorio et al., 2018) (Figure 2A). Such work has presented strong evidence for life-long multilineage output from adult human HSCs. Moreover, it has been demonstrated that the human HSC pool undergoes dynamic changes throughout development, where the number of HSCs steadily increases in early life to reach a plateau by adulthood. *Via* the analysis of spontaneous nuclear DNA mutations, it was estimated that the number of HSCs falls within the range of 50,000–200,000 in an adult individual, with active HSC contributions to the granulocyte and B lymphocyte lineages but to a lesser extent to the T cell lineage (Lee-Six et al., 2018). An evident limitation with these technologies is the large size of the nuclear genome that needs to be surveyed to identify the rather infrequent somatic mutations, which in essence restricts the amount of traceable cells and make such approaches costly to implement routinely (Lee-Six et al., 2018). Emerging methodology that quantifies mitochondrial (mt) DNA mutations appears as one potential solution to this, which has the added benefit of being compatible with simultaneous transcriptome and chromatin accessibility profiling (Ludwig et al., 2019). Clonal tracking based on mtDNA heteroplasmy was successfully implemented to dissect clonal relationships in between human HSPCs (Lareau et al., 2021). Notably, and in line with studies employing nuclear DNA mutations as traceable endogenous barcodes (Lee-Six et al., 2018; Osorio et al., 2018), the use of mtDNA mutation patterns suggested an active contribution of a large pool of HSPCs to steady-state hematopoiesis (Lareau et al., 2021).

Additional evidence for human HSCs multipotency and self-renewal has been derived from clinical BM transplantation (BMT) studies. HSPC-based gene therapy (GT) in combination with transplantation represents a therapeutic avenue to cure inherited genetic blood cell disorders. Analogous to cellular barcoding, GT employs *ex vivo* gene transfer through stable integration of viral vectors in HSPCs. As the viral vectors integrate randomly in the genome, they in addition to the therapeutic gene also create stable marks (integration sites; IS) that are passed to daughter cells during differentiation (Ferrari et al., 2021). Thereby, IS analysis in BM and mature blood cells allows for determination of clonality and survival of HSPCs following transplantation (Figure 2B).

Initial IS studies in patients with SCID established sustained production of T lymphocytes but with limited long-term engraftment of the myeloid and B lineages (Hacein-Bey-Abina et al., 2010), despite low-level donor-chimerism in these lineages during the first years following transplantation (Hacein-Bey-Abina et al., 2002). While providing some evidence of lymphoid-biased hematopoiesis, several factors can contribute to this outcome. Transplantation into patients lacking (functional) lymphoid cells means that gene-corrected clones are privileged to efficiently repopulate the lymphoid compartment and may in turn lead to imbalanced clonal dynamics and limited reconstitution of other blood lineages. Furthermore, SCID patients that undergo BMT typically do not require intensive conditioning. As conditioning also empties the BM niches for the transplanted cells, it is likely that the limited available niches in the BM of unconditioned SCID patients can restrict effective engraftment of multilineage reconstituting HSPC clones. Support for this comes from a study of adenosine deaminase (ADA) SCID patients, where stable multilineage engraftment long-term following BMT was observed only in a patient who received conditioning (Cancrini et al., 2010).

Longitudinal studies in patients with other inherited disorders have provided further insights into the nature of human hematopoiesis. These have demonstrated multilineage output from transplanted HSPCs, as indicated by multiple shared IS that could be found in the myeloid and lymphoid lineages (Cartier et al., 2009; Aiuti et al., 2013; Biffi et al., 2013; Biasco et al., 2016; Scala et al., 2018). This associated with polyclonal and stable IS patterns long-term after transplantation, although increasing evidence suggests that along with multipotent clones, lineage restricted HSPCs are also involved in the daily maintenance of human hematopoiesis (Scala et al., 2018; Six et al., 2020). This is in line with both previous gene expression profiling studies (Velten et al., 2017) and studies on mouse hematopoiesis (Dykstra et al., 2007; Yamamoto et al., 2013). Finally, post-transplantation hematopoiesis in humans was shown to be sustained by distinct HSPC subsets over time. Similar to observations in non-human primates (Kim et al., 2014; Wu et al., 2014; Koelle et al., 2017), one progenitor class sustained hematopoiesis for ∼6–12 months, which was subsequently replaced by more long-term and stably contributing HSC clones around 1 year after transplantation (Scala et al., 2018).

Altogether, existing data imply that human hematopoiesis, similar to the mouse, is maintained by a pool of multipotent HSPCs throughout life. This does not preclude the existence of HSC-like clones with lineage-biased differential potential, nor more long-lived HSPCs, which could serve to more readily respond to specific challenges when needed.

## Discussion

From novel reporter mouse models and advances in high-throughput sequencing, we know today that both murine and human native hematopoiesis is dynamically orchestrated by a heterogenous pool of primitive HSPCs. Hematopoiesis associates with a low daily input from HSCs but a higher proliferative activity of different intermediate progenitor subsets. Hence, native hematopoiesis is highly polyclonal, which presumably allows for a high flexibility depending on organismal demands. In contrast, hematopoietic stress and external perturbations instantly evoke oligoclonal responses from HSPCs to counteract cell loss and allow for return to homeostasis. In the transplantation setting, this is manifested by distinct phases of reconstitution, which primarily rely on the activity of progenitors downstream of HSCs, and which is followed by the contribution from more long-lived multipotent clones. However, what remains unknown is to what extent the clonal behavior of individual HSPCs is pre-determined at the molecular level, and whether or not it is possible to induce specific HSC programs by genetic or pharmacological interventions. Additional studies using novel lineage tracing tools, advanced molecular profiling techniques and newer *in vitro* culture systems are likely to provide answers to these questions in a foreseeable future.
